# Chemical Characterization, Sensory Evaluation, and Biological Activity in Neuronal Cells of Essential Oils (Rose, Eucalyptus, Lemon, and Clove) Used for Olfactory Training

**DOI:** 10.3390/molecules30173591

**Published:** 2025-09-02

**Authors:** Antonella Rosa, Franca Piras, Alessandra Piras, Silva Porcedda, Valeria Sogos, Carla Masala

**Affiliations:** 1 Department of Biomedical Sciences, University of Cagliari, Cittadella Universitaria, SS 554, km 4.5, 09042 Monserrato, CA, Italy; fpiras@unica.it (F.P.); sogos@unica.it (V.S.); cmasala@unica.it (C.M.); 2 Department of Chemical and Geological Sciences, University of Cagliari, Cittadella Universitaria, SP 8, Monserrato-Sestu km 0.700, 09042 Monserrato, CA, Italy; apiras@unica.it (A.P.); porcedda@unica.it (S.P.)

**Keywords:** essential oils, olfactory training, rose, eucalyptus, lemon, clove, volatile components, SH-SY5Y cells, cytotoxicity

## Abstract

Essential oils (EOs) are natural mixtures of volatile compounds characterized by beneficial pharmacological effects. The repeated inhalation of EOs in olfactory training (OT) has been demonstrated to improve the sense of smell in patients with olfactory deficits. We conducted a conjunct evaluation of the chemical composition, sensory profile, and bioactivity in cell models of commercial EOs of rose (EO1), eucalyptus (EO2), lemon (EO3), and clove (EO4) used for OT (StimuScent^®^, Dos Medical, Sense Trading BV, Groningen, The Netherlands). Citronellol, 1,8-cineole, limonene, and eugenol emerged as the most abundant volatile compounds in EO1, EO2, EO3, and EO4, respectively, by GC-MS analysis. Some differences emerged (using a Likert-type scale) in the perception of EO’s odor dimensions (pleasantness, intensity, and familiarity in subjects with hyposmia (n = 8) compared to controls (n = 22). Cytotoxicity assays (24 h of incubation) demonstrated the anticancer effects of EOs (5–100 μg/mL) on SH-SY5Y neuroblastoma cells (the order of potency was EO3 > EO4 > EO2 > EO1), while all EOs showed lower effects on the viability/morphology of human skin HaCaT keratinocytes. SH-SY5Y cancer cells grown for six days with different EOs (at 50 μg/mL) showed evident signs of toxicity and apoptosis. Marked changes in cell morphology (structure/number of processes) were evidenced in clove EO-treated cells. EO’s sensory properties/bioactivity were also related to the in silico physicochemical/pharmacokinetic properties of the main EO components. Our results provide new insights into a more targeted EO application for OT.

## 1. Introduction

Essential oils (EOs) are naturally occurring complex liquid mixtures of volatile, aromatic, and lipophilic compounds, synthesized as secondary metabolites by plants for defense, growth control, and communication/interaction with other species or plants [1,2,3,4,5]. EOs are a complex mixture of terpenoids, phenols, aldehydes, ketones, ethers, epoxides, and other compounds that can be extracted from various parts of plants, including flowers, leaves, seeds, peels, branches, bark, wood, roots, gums, or oily resins [5]. However, two or three components are usually present in large proportions (20–70%) [1]. Various techniques are employed for EO extraction, from traditional methods such as steam or hydro-distillation, solvent extraction, maceration, cold pressing, and enfleurage, to those that are more recent and promising, including supercritical fluid extraction, microwave-assisted extraction, and ultrasound-assisted extraction [2,4,6,7].

EOs are utilized across various industries, including perfumery, aromatherapy, cosmetics, food flavoring and preservation, and natural pharmacological treatments, due to their unique chemical compositions and potential health benefits [3,6,7]. These EOs have been considered attractive for their wide variety of bioactivities, including anti-inflammatory [1,3,4], antioxidant [1,2,3,4,8], antimicrobial [1,2,3,4], antiviral [1,2,3,4], antidiabetic [2], analgesic [2,4], and anticancer properties [1,2,4,7,9,10], among others.

A growing number of studies have documented the efficacy of EOs and their chemical constituents as antitumor agents in cancer cell lines or animal models [1,2,7,9,10]. The potent anticancer benefits of EOs and their constituents are the results of multiple pathways and mechanisms involving induction of apoptosis, cytotoxicity, antiproliferative activity, cell cycle arrest, loss of key organelles function, DNA repair modulation, pro-oxidant effects, and effects on tumor suppressor proteins, transcription factors, and detoxification enzymes [1,2,7,9,10,11]. Moreover, several studies have highlighted the beneficial EO effects on the central nervous system (CNS) [12,13] and their role as promising neuroinflammation modulators in neurodegenerative diseases [13,14,15]. Due to their lipophilic nature and low molecular weights, EO constituents can cross cell membranes altering the phospholipid layers, increasing membrane fluidity, and interfering with biological processes at cellular and multicellular levels via interaction with various biological targets [1].

The term “aromatherapy” collectively refers to the use of EOs by oral administration (ingestion), topical application (skin absorption), and inhalation through the nose [5,12,16,17]. However, inhalation by the olfactory system is generally considered as the fastest and easiest method of EO administration [12]. Numerous studies have highlighted the beneficial pharmacological effects of EO inhalation on the CNS [5,12,13,14,16]. The inhalation of EOs stimulates neural pathways which are involved in emotion and memory, inducing antistress, antianxiety, analgesic, cognitive, and autonomic effects, and improving memory deficit [12,17]. The olfactory pathway involves a direct route from olfactory receptors in the nasal cavity (olfactory epithelium) to the olfactory bulb, then to the olfactory cortex and, importantly, to the limbic system (including the amygdala and hippocampus) [12,17,18].

Olfactory training (OT) involves the repetitive and mindful smelling/inhalation of specific EOs over a period, typically twice daily for several months (from 3 to 8 months), which helps to stimulate/restore damaged or underused neural pathways in the olfactory system [17,19,20]. This scientifically supported method has been demonstrated to restore or enhance the sense of smell after conditions like viral infections (e.g., SARS-CoV-2 virus), trauma, neurological disorders, chronic rhinitis and sinusitis, and cancer treatments [17,19,20]. Some commonly used oils in OT include the EOs of lavender, peppermint, lemon, eucalyptus, clove, and rose, chosen for their distinct and potent aromas, as well as their bioactive compounds that may support neurological and immune health [17,19]. The accepted protocol for OT consists of intensively smelling four different EOs (in a random order) twice a day for a period of 12 weeks. Four EOs typically used in the OT (4-item-OT) are those of rose, eucalyptus, lemon, and clove [19].

Starting from all these considerations, the objective of the present research is to provide a wide characterization of commercial rose (*Rosa damascena*), eucalyptus (*Eucalyptus globulus*), lemon (*Citrus limon*), and clove (*Syzygium aromaticum* or *Eugenia caryophyllus*) EOs included in the StimuScent^®^ smell training kit-SET 1, a kit normally used in clinical practice for the improvement of the sense of smell in patients with olfactory deficits [17,19,20], through the conjunct evaluation of their chemical composition, sensory profile, and bioactivity in cell models.

The volatile component profile of EOs was determined by gas chromatography coupled with mass spectrometry (GC-MS). The olfactory dimensions (odor pleasantness, intensity, and familiarity) of EOs were estimated in non-trained subjects (including healthy participants and participants with hyposmia) using the hedonic scale method (Likert scale) [21,22]. Then, EOs’ effects on cell viability/morphology were tested after incubation (24 h and 6 days) in SH-SY5Y cancer cells, a human neuroblastoma cell line extensively used in neuroscience research [23,24], and in human skin HaCaT keratinocytes, a skin cell model amply used to assess the toxicity/biocompatibility of natural extracts/compounds [25,26].

Moreover, the sensory properties/bioactivity of EOs were related to the in silico physicochemical/pharmacokinetic properties of their main volatile compounds, assessed using the PubChem database [27] and the freely accessible web tools SwissADME [28] and pkCSM-pharmacokinetics [29].

The concerted determination of chemical composition, sensory properties, bioactivity, and physicochemical/pharmacokinetic properties will provide new insights on rose, eucalyptus, lemon, and clove EOs useful for a more targeted OT application.

## 2. Results

### 2.1. Chemical Composition of EOs by GC-MS Analysis

**Figure 1:**
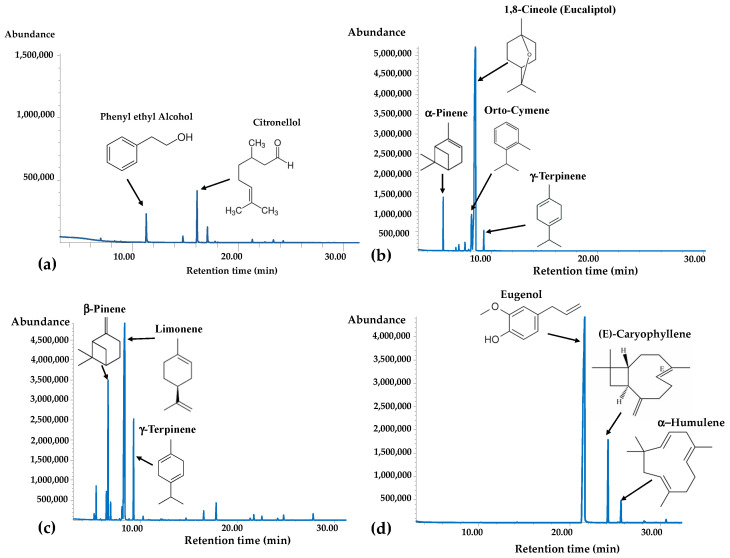
Chromatograms, obtained by GC/MS technique on an HP-5MS capillary column, of EO1 (**a**), EO2 (**b**), EO3 (**c**), and EO4 (**d**) with the indication of the main volatile components.

The volatile compounds of EOs from rose (EO1), eucalyptus (EO2), lemon (EO3), and clove (EO4), contained in a smell training kit (StimuScent^®^), were analyzed by GC/MS. Figure 1 shows the chromatographic profile (within the separation time range 0–30 min) of EO1 (Figure 1a), EO2 (Figure 1b), EO3 (Figure 1c), and EO4 (Figure 1d) by GC/MS technique with the indication of the main identified volatile compounds, while full chromatograms are reported in Figure S1. The chemical composition (expressed as % *w*/*w*) of the four products is reported in Table 1.

Chemical analysis revealed that EO1 (rose) had a very high content of the ester isopropyl hexadecanoate (94.8% of total components), an odorless organic compound used as a diluent in this commercial product, with no effect on the smell quality of the product.

Citronellol (2.3%), phenyl ethyl alcohol (1.4%), and geraniol (0.8%) were identified as the main rose volatile components. Other EO1 components, with relatively small amounts, were dihydro citronellol (0.3%), citronellyl acetate (0.1%), linalol butanoate (0.1%), and myrcene (0.1%).

The monoterpene cyclic ether 1,8-cineole (82.7%) and the monoterpene hydrocarbons para-cymene (7.9%) and α-pinene (4.9%) represented the most abundant volatile compounds found in EO2 (eucalyptus), followed by small amounts of γ-terpinene (2.3%) and α-phellandrene (1%).

GC-MS analysis allowed us to identify 17 compounds in EO3 (lemon) and among them, limonene was found to be the major component, accounting for 55.9%, followed by β-pinene (15.5%) and γ-terpinene (11.1%). Other components, with relatively small amounts, were α-pinene (2.9%), sabinene (2.8%), para-cymene (2.3%), geranial (2.1%), and myrcene (1.6%).

EO4 (clove) was characterized by high amounts of the phenylpropanoid eugenol (86.6%), followed by (*E*)-caryophyllene (10.2%) and α-humulene (2.5%), and small amounts of δ-amorfene, *trans*-calamelene, and (*Z*)-dihydro-apofarnesol.

### 2.2. Assessment of EOs Sensory Properties

**Figure 2:**
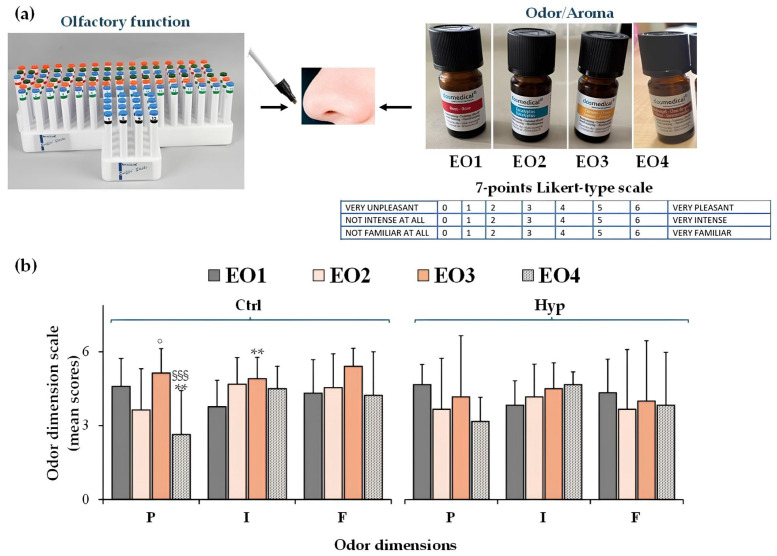
Sniffin’ Sticks test for the assessment of olfactory function and the hedonic Likert-type scale (7-points, from 0 to 6) used to assess odor dimensions (pleasantness, intensity, and familiarity) of EO1 (rose), EO2 (eucalyptus), EO3 (lemon) and EO4 (clove) (**a**). Ratings of odor pleasantness (P), intensity (I), and familiarity (F) dimensions of EO1, EO2. EO3, and EO4 measured in healthy controls (Ctrl, n = 22) and participants with hyposmia (Hyp, n = 8) (**b**). Data are presented as mean values and standard deviations. Significant differences between groups (One-way ANOVA adjusted with the Bonferroni Multiple Comparisons Test): ** = *p* < 0.01 versus EO1; ° = *p* < 0.05 versus EO2; ^§§§^ = *p* < 0.001 versus EO3. No significant differences were determined for subjects with hyposmia (Hyp) versus controls (Ctrl).

Thirty participants (untrained panelists) were enrolled to assess the sensory properties of EO1, EO2, EO3, and EO4. Preliminary, the Sniffin’ Sticks test was used for the assessment of the olfactory function of participants (Figure 2a).

This test, which consists of pen-like odor-dispensing devices for the determination of odor threshold (OThr), odor discrimination (ODi), and odor identification (OId) [21,22], allowed us to identify eight subjects with hyposmia among participants.

Table 2 shows mean values ± standard deviation (SD) measured for age, sex, weight, height, body mass index (BMI), OThr, ODi, OId, and TDI score in healthy controls (Ctrl, n = 22) and participants with hyposmia (Hyp, n = 8).

In this population, no significant differences were found in age, weight, height, and BMI between subjects with hyposmia compared to controls, while participants with hyposmia showed a marked significant decrease in olfactory function. In subjects with hyposmia, mean scores were significantly lower than in controls for OThr (*p* < 0.001), ODi (*p* < 0.001), OId (*p* < 0.001), and TDI score (*p* < 0.001) (Table 2).

Then, a 7-point hedonic Likert-type scale (Figure 2a) was used to evaluate EOs’ odor dimensions (pleasantness, intensity, and familiarity) in the two groups of participants to evidence differences in the sensory perception of EOs used for OT in participants with chemosensory deficits versus healthy controls [21,22].

Figure 2b shows the ratings of odor pleasantness (P), intensity (I), and familiarity (F) dimensions measured for the odor of EO1 (rose), EO2 (eucalyptus), EO3 (lemon), and EO4 (clove) in healthy participants (Ctrl) and participants with hyposmia (Hyp).

Significant differences were observed in odor pleasantness and intensity among EOs in healthy participants. In this group, the EO3 odor emerged as the most pleasant (EO3 > EO1 > EO2 > EO4), intense (EO3 > EO2 > EO4 > EO1), and familiar (EO3 > EO2 > EO4 > EO1), while EO4 and EO1 odors were perceived as the least pleasant and intense, respectively.

Positive correlations were found between odor pleasantness/familiarity (r = 0.7192) and intensity/familiarity (r = 0.6655) of tested EOs in healthy participants.

In participants with hyposmia, the EO1 odor emerged as the most pleasant (EO1 > EO3 > EO2 > EO4), while EO4 odor was perceived as the most intense (EO4 > EO3 > EO2 > EO1). As observed in healthy subjects, a positive correlation (r = 0.8315) was found between EO odor pleasantness/familiarity in participants with hyposmia, while a negative correlation (r = −0.7569) was found between odor pleasantness/intensity of tested EOs.

Interestingly, certain slight differences were observed in the sensory perception of EO odor pleasantness, intensity, and familiarity dimensions between participants with hyposmia compared to healthy controls, despite noticeable differences observed in olfactory functions between the two groups.

Participants also provided a subjective description of the odor (aroma) of the four products and the results obtained for healthy controls and participants with hyposmia are listed in Table 3. Descriptors of the odor of EOs’ main constituents, identified by GC-MS, from literature data [27] are also reported in Table 3.

Regarding EOs’ odor (aroma), some participants of both groups recognized the exact essential oil. In general, healthy controls furnished more sensory descriptors than subjects with hyposmia.

Despite the presence of a high quantity of isopropyl hexadecanoate (an odorless diluent) [27], the rose scent was individuated by both groups, essentially attributable to EO1 components like citronellol, characterized by a fresh rosy odor, and phenyl ethyl alcohol, which possesses a rose-like odor [27].

Healthy participants described the EO2 aroma as eucalyptus, fennel, licorice, anise, herbs, mint, menthol, lavender, camphor, tiger balm, tea tree oil, and peppermint, due to the characteristic fresh minty, woody, and camphoraceous aroma of this EO [27]. Participants with hyposmia, in addition to eucalyptus, described the EO2 aroma as unpleasant, aromatic/minty, and medicinal.

Lemon EO (EO3) for both groups was primarily characterized by a citrus-like aroma, influenced by aromatic terpenes like limonene. Some participants with hyposmia described EO3 as sweet and fresh.

Both healthy participants and those with hyposmia indicated a clove-like aroma for EO4 (clove); however, various sensory descriptors (incense, cinnamon, smoked, spicy, herbs, leather, and eucalyptus) were used by healthy participants for EO4 odor.

### 2.3. Effect of EOs on Cell Viability After 24 h of Incubation (MTT Assay)

The MTT assay was utilized to assess the cytotoxic effects of EO1, EO2, EO3, and EO4 on SH-SY5Y neuroblastoma cells.

**Figure 3:**
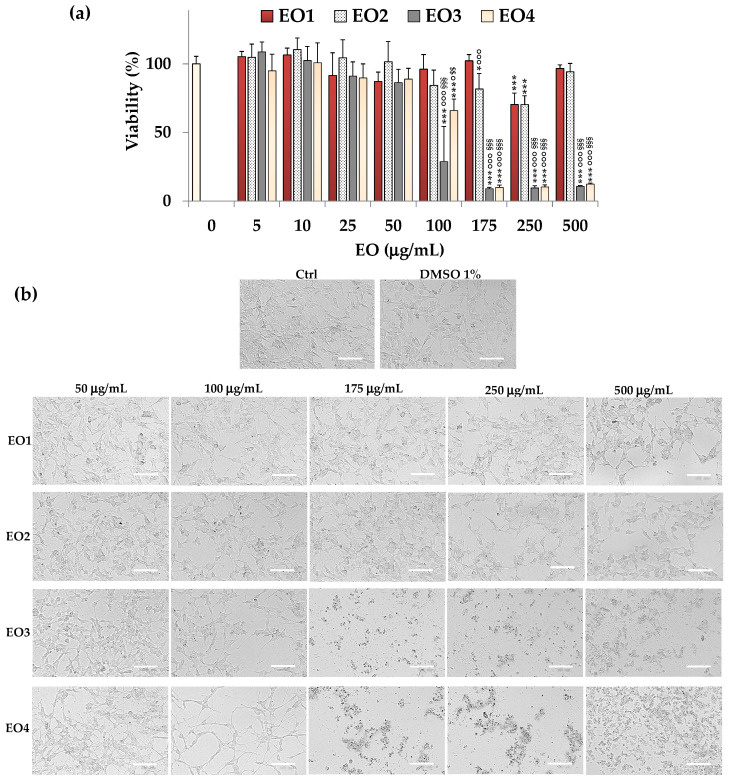
(**a**) Values of viability (MTT assay) measured in control (untreated) cancer SH-SY5Y cells (0, 100% viability) and cells incubated for 24 h with different amounts (from 5 to 500 μg/mL) of EO1, EO2, EO3, and EO4. Three independent experiments were performed, involving four analyses for each sample (n = 12). One-way ANOVA and the Bonferroni Multiple Comparisons Test were used to assess the statistical significance of differences. For each series: *** = *p* < 0.001, * = *p* < 0.05 versus control cells (0). For each concentration: °°° = *p* < 0.001, ° = *p* < 0.05 versus EO1; ^§§§^ = *p* < 0.001 versus EO2; ^$$^ = *p* < 0.01 versus EO3. (**b**) Phase contrast images of SH-SY5Y control cells (Ctrl), and cells incubated for 24 h with DMSO (1% *v*/*v*) and EO1, EO2, EO3, and EO4 (50, 100, 175, 250, and 500 μg/mL). Bar = 100 μm.

The viability values (reported as a percentage of control cells, 100% of viability) of cells treated for 24 h with various amounts (ranging from 5 to 500 μg/mL) of the four EOs are shown in Figure 3.

The tested EOs exhibited different cytotoxic effects in cancer cells, with the following order of potency: EO3 > EO4 > EO2 > EO1.

After 24 h of incubation, lemon EO (EO3) significantly reduced cancer cell viability (71%, *p* < 0.001 versus control cells) at 100 μg/mL; moreover, it induced a cell growth inhibition of nearly 90% (*p* < 0.001) in the range of 175–500 μg/mL.

EO4 was not significantly cytotoxic in cancer SH-SY5Y cells from 5 to 50 μg/mL (5–11% of viability reduction), whereas it induced a significant (*p* < 0.001 versus control cells) dose-dependent cell growth inhibition in the concentration range 100–500 μg/mL (34–90% of viability reduction).

A clear inhibition (16–30%) of cancer cell growth was evident after 24 h-treatment with 100–250 μg/mL of EO2.

Cells treated for 24 h with EO1 did not show a significant decrease in viability versus control cells in the range of 5–175 μg/mL, while a significant inhibition (*p* < 0.001) of cancer cell growth (30%) was observed at 250 μg/mL.

The vehicle DMSO did not affect cell viability at any of the tested amounts (0.125–1.25% *v*/*v*).

The IC_50_ values (the EO concentration that decreases the cell viability to 50%) of EO3 (lemon) and EO4 (clove) were 77.5 μg/mL and 120.5 μg/mL, respectively, after 24 h incubation in neuroblastoma SH-SY5Y cells. The IC_50_ value was not determined for EO1 and EO2 because it exceeded the maximum percentage (1%) of DMSO tolerated in SH-SY5Y cells.

The treatment with EO1, EO2, EO3, and EO4 induced marked changes in cell morphology versus untreated cells, as evidenced by phase contrast microscopy. Phase contrast images of SH-SY5Y control (untreated) cells and cancer cells treated for 24 h with 50–500 μg/mL of EOs are shown in Figure 3b. Untreated neuroblastoma cells were fusiform and characterized by the presence of short, branched processes. Phase contrast microscopy did not show significant changes in cell morphology or density of SH-SY5Y cells treated with 1% *v*/*v* DMSO (maximal dose used to dissolve EOs) versus control cells.

The treatment (24 h) with EO1 and EO2 only induced a decrease in the total cell number, evident at 250 μg/mL, without effects on cell morphology.

The treatment with EO3 at 100 μg/mL induced a reduction in the total cell number, and a dose-dependent increase in the number of cells with a reduced size/ rounded morphology (apoptotic cells) was observed from 50 μg/mL. Only rounded cells and cell debris were evidenced in EO3-treated cells in the range 175–500 μg/mL.

Areas with a decreased cell density were observed in cancer SH-SY5Y treated with 100 μg/mL of EO4, suggesting a reduction in proliferation rate. Moreover, the cells displayed a more fusiform (spindle-like) shape and increased connections with other cells (branched neurite projections), suggesting that treated cells were probably undergoing differentiation into a neuronal phenotype [23,24]. A great number of rounded (apoptotic) cells and cell debris were observed from 175 μg/mL of EO4.

HaCaT keratinocytes, a normal human skin cell line amply used to assess the biocompatibility of herbal extracts and isolated natural compounds [25,26], were then utilized to evaluate the cytotoxic effect of EOs.

**Figure 4:**
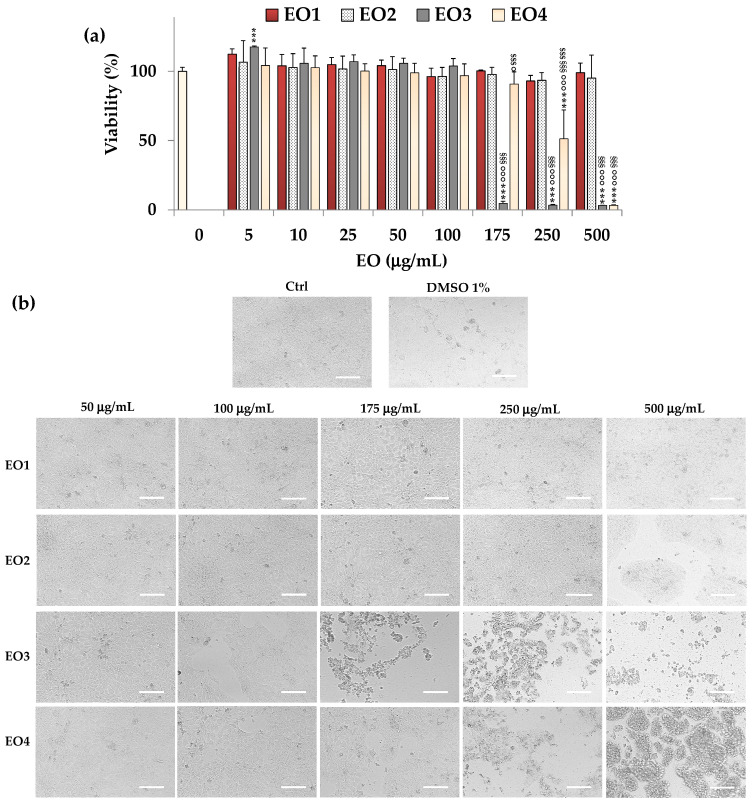
(**a**) Values of viability (MTT assay) measured in control (untreated) HaCaT cells (0, 100% viability) and cells incubated for 24 h with different amounts (5–500 μg/mL) of EO1, EO2, EO3, and EO4. Three independent experiments are performed, involving four analyses for each sample (n = 12). One-way ANOVA and the Bonferroni Multiple Comparisons Test were used to assess the statistical significance of differences. For each series: *** = *p* < 0.001 versus control cells (0). For each series: *** = *p* < 0.001 versus the respective control (0). For each concentration: °°° = *p* < 0.001, ° = *p* < 0.05 versus EO1; ^§§§^ = *p* < 0.001 versus EO2; ^$$$^ = *p* < 0.001 versus EO3. (**b**) Phase contrast images of HaCaT control cells (Ctrl), and cells incubated for 24 h with DMSO (1% *v*/*v*) and EO1, EO2, EO3, and EO4 (50, 100, 175, 250, and 500 μg/mL). Bar = 100 μm.

Figure 4a displays viability values (as % of the control) measured by MTT assay in HaCaT cells after the 24 h-treatment with 5–500 μg/mL of EOs, while the phase contrast images of HaCaT cells incubated with 50–500 μg/mL of EOs are reported in Figure 4b.

All tested concentrations of EO1 and EO2 did not induce marked changes in HaCaT cell viability, demonstrating a more selective toxicity towards cancer cells than normal cells at high concentrations.

IC_50_ values of 138.8 μg/mL and 255.5 μg/mL were measured for EO3 and EO4, respectively, in healthy human HaCaT keratinocytes after 24 h of incubation, highlighting a lower cytotoxic effect than in cancer SH-SY5Y cells. The vehicle DMSO was not toxic in HaCaT cells, and the cell viability at the maximal tested dose (1%) was 91%.

Cells treated for 24 h with EO1 and EO2 evidenced, at 50–500 μg/mL, morphological traits (spindle-shaped and adherent cells) similar to those of control cells (Figure 4b). The presence of rounded cells was observed from 175 μg/mL and 250 μg/mL for EO3 and EO4, respectively, evidencing the lower cytotoxic effects of both EOs on skin keratinocytes than on cancer cells.

### 2.4. Bioactivity of EOs on SH-SY5Y Cells After 6 Days of Incubation

A different effect on cell growth and morphology was observed in neuroblastoma cells treated for 24 h with various EOs. Therefore, the effect of the four EOs on cancer SH-SY5Y cell viability, apoptosis, and morphology was compared after a long period of incubation (6 days) with a non-toxic dose. The concentration of 50 mg/mL was selected based on preliminary data on SH-SY5Y cell viability/morphology after 24 h of incubation with EOs (as reported in Figure 3).

**Figure 5:**
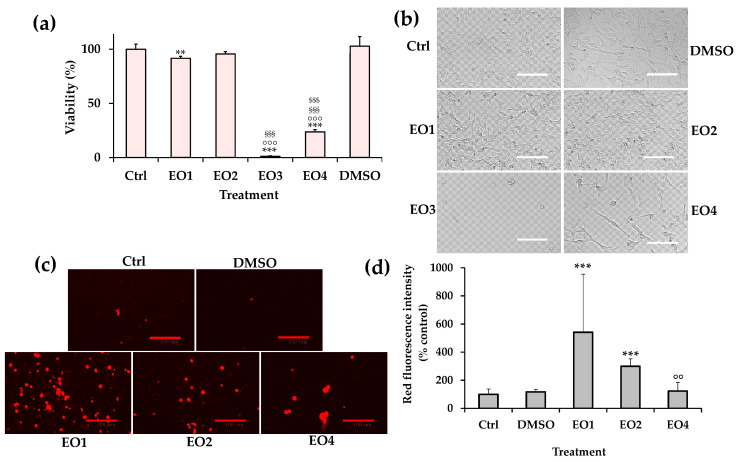
Viability values (expressed as % control, MTT assay) (**a**), representative phase contrast images before MTT assay (**b**), fluorescence red emission images, by propidium iodide (PI) assay (**c**), and intensity of PI red emission fluorescence (expressed as % control) (**d**), obtained in SH-SY5Y control cells (untreated, Ctrl) and cells incubated for 6 days with 50 μg/mL of EOs and the corresponding vehicle dose (DMSO 0.1% *v*/*v*). Data are presented as mean and standard deviation (SD) of three experiments involving triplicate analyses for each sample (n = 9). The statistical significance of differences was assessed by One-way ANOVA, followed by the Bonferroni Multiple Comparisons Test. For each series: *** = *p* < 0.001, ** = *p* < 0.01 versus the respective control cells (Ctrl); °°° = *p* < 0.001, °° = *p* < 0.01 versus EO1; ^§§§^ = *p* < 0.001 versus EO2. ^$$$^ = *p* < 0.001 versus EO3. Bar = 100 μm.

Figure 5 shows the viability values (expressed as % of control cells) (Figure 5a) and phase contrast images (Figure 5b), before the MTT assay, measured in control (untreated) cells (100% viability) and cells treated for 6 days with 50 μg/mL of EOs and the corresponding amount of DMSO (0.1% *v*/*v*).

According to 24 h of incubation, a different cytotoxic effect was detected in cancer cells treated for 6 days with various EOs at the dose of 50 μg/mL, and the order of potency was EO3 > EO4 > EO1 ≅ EO2. The treatment with lemon EO (EO3) induced a marked viability reduction (nearly 99%, *p* < 0.001 versus control cells), and only scarce rounded and floating cells were observed by phase contrast microscopy.

EO1 and EO2 induced a slight reduction in cancer cell viability (less than 10%) and phase contrast microscopy allowed to highlight a rise in the number of rounded (apoptotic) cells.

EO4 significantly (*p* < 0.001) decreased (76%) viability in treated neuroblastoma cells in comparison with control cells. Phase contrast image (Figure 5b) evidenced a reduced cell number and the presence of apoptotic cells. Cells with a more fusiform shape were also observed, probably attributable to a differentiation process [23,24].

Therefore, cancer SH-SY5Y cells were stained with propidium iodide (PI), a DNA-binding fluorescent dye (red) able to evidence the effect of EOs on apoptosis/cell death [23,24]. The red emission images obtained, after 2 h of incubation with PI dye, for SH-SY5Y control cells and cells grown for 6 days in the presence of EO1, EO2, and EO4 (50 μg/mL), and 0.1% *v*/*v* DMSO are shown in Figure 5c, whereas quantitative values of cell PI fluorescence intensity (expressed as % control cells) are reported in Figure 5d.

Control cells and DMSO-treated cells showed a low basal level of red fluorescence. Cells treated with EO1 and EO2 displayed a significant increase in red fluorescence intensity (542% and 299% versus control cells for EO1 and EO2, respectively), indicating a late apoptosis/necrosis induction. A slight increase in red fluorescence was also observed in EO4-treated cells, while the high mortality rate present in EO3-treated cells did not consent to the evaluation of apoptosis after 6 days of incubation.

Then, the immunocytochemical staining of human cancer SH-SY5Y cells for neuronal markers such as neurofilaments (NF) was performed after 6 days of treatment in the presence of EO1, EO2, and EO4 at a dose of 50 μg/mL to evidence potential effects of extracts in altering the NF structural organization.

**Figure 6:**
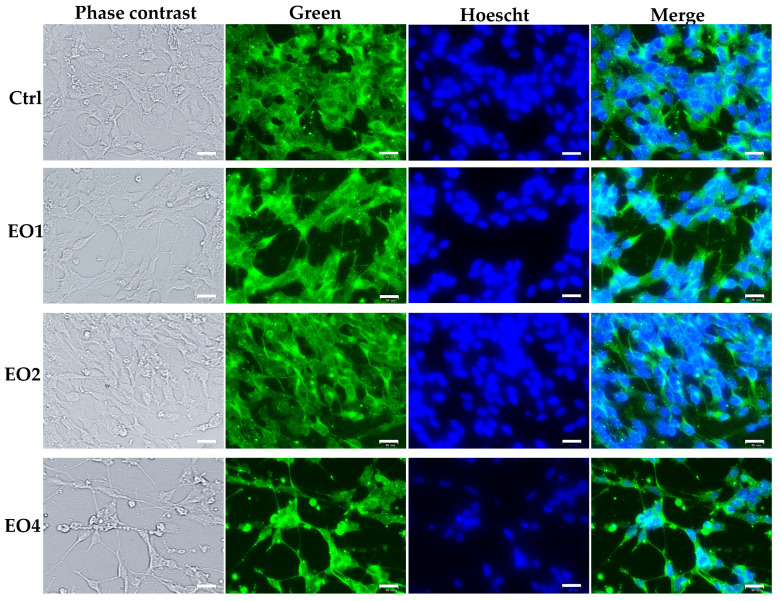
Immunocytochemical staining for neurofilaments (NF) (green) on human neuroblastoma SH-SY5Y cells (untreated, Ctrl) and cells treated for 6 days with EO1, EO2, and EO4 at a dose of 50 μg/mL. Nuclei are stained with Hoechst dye 33258 (blue). Merged images (green and blue) are displayed in the last column, while phase contrast images (gray) are reported in the first column. Bar = 25 μm.

Figure 6 shows double staining for NF (green) and Hoechst dye 33258 (nuclei) of untreated neuroblastoma cells and cells treated for 6 days with EOs.

In control neuroblastoma cells, green fluorescently labeled neurofilaments were observed to be distributed throughout the cell, particularly concentrated in the cell body and extending into neurites (processes). Similar distribution was observed in cells treated with EO1 and EO2.

A marked increase in NF green fluorescence levels was visually observed when neuroblastoma cells were treated with clove EO (EO4), especially in the neurites (processes), which appeared longer and more intense, highlighting a potential effect on cell differentiation to a neuronal phenotype.

### 2.5. In Silico Evaluation of Physicochemical and Pharmacokinetic Properties of the Main Volatile Components of EOs

The canonical SMILES (Simplified Molecular-Input Line Entry Specification nomenclature) [32] of EOs’ main volatile compounds (citronellol, 1,8-cineole, limonene, and eugenol) were obtained by the PubChem web database [27] (Table S1).

This database also provided several physicochemical properties of selected compounds, including molecular weight (MW), rotatable bond count (RBC), hydrogen bond donor count (HBDC), hydrogen bond acceptor count (HBAC), lipophilicity (XLogP3-AA), complexity, topological polar surface area (TPSA), and vapor pressure (VP) (Table S2).

The canonical SMILES of selected compounds were entered into the freely accessible web tools SwissADME [28] and pkCSM-pharmacokinetics [29] to obtain important in silico pharmacokinetic properties, such as human gastrointestinal absorption (HIA), blood–brain barrier (BBB) permeability, the ability to be a substrate of glycoprotein P (P-gp), skin permeation, and central nervous system (CNS) permeability (Table S2) [32,33,34].

The Bioavailability Radars obtained by SwissADME [28] for citronellol, 1,8-cineole, limonene, and eugenol are reported in Figure S2. In this graphical representation, the pink area indicates the optimal range for various properties that qualify a successful drug (drug-likeness) [28,33]. This “optimal range” indicates properties desirable for a good oral bioavailability, including lipophilicity (XLogGP3 between −0.7 and +5.0), size (MW between 150 and 500 g/mol), polarity (TPSA between 20 and 130 Å^2^), water solubility (log S not higher than 6), saturation, and flexibility (no more than 9 rotatable bonds) [28,33]. All selected compounds emerged as orally bioavailable, mostly entering into the pink area [28,33]. Percentages of compounds predicted to be absorbed through the human intestine (HIA%), computed by pkCSM-pharmacokinetics, were 92.83%, 96.505%, 95.898%, and 92.041% for citronellol, 1,8-cineole, limonene, and eugenol, respectively, qualifying the ability of all compounds to cross cell membranes [29,34].

In the “BOILED-Egg” representation (Figure S3), obtained by SwissADME [28], all selected volatile compounds appeared as a red point in the yellow region (yolk), indicating a high probability of blood–brain barrier (BBB) penetration [28,33].

## 3. Discussion

The sense of smell is recognized as one of the most important senses in humans and a key determinant of behavior [18]. The olfactory function has a crucial role in human life for avoiding potentially dangerous compounds and for detecting spoiled and rotting foods [22,35,36]. Olfactory disorders often decrease the quality of life [19,35].

EOs, complex natural mixtures of volatile, lipophilic, and odoriferous substances commonly found in aromatic plants, have been widely investigated for their therapeutic potential in various pathologies, including cancer and neurodegenerative diseases [1,2,3,4,5,7,9,10,11,14,15]. EOs have specific beneficial effects on CNS affecting mood, memory, and cognitive function [5]. EOs can produce a variety of CNS targeted pharmacological effects such as neuroprotection and anxiolytic, antidepressant, anticonvulsant, analgesic, and sedative effects [5,12,13]. Various EOs have shown promising results in many in vitro and in vivo preclinical models of neurodegenerative disorders, counteracting oxidative stress and neuroinflammation and rescue from neuronal death and neurodegeneration [14,15].

Moreover, several EOs showed anticancer activity in neuroblastoma and glioblastoma [9,11].

The inhalation of EOs has been demonstrated to resensitize the neural pathways that allow the identification of aroma and flavor [17]. OT, the regular, systematic exposure to a set of EOs, is used for the rehabilitation of the sense of smell in clinical practice and is based on the concept of training olfactory sensory neurons to relearn and distinguish olfactory stimuli [17,19,20,36]. Rose, eucalyptus, lemon, and clove EOs are amply used for OT in clinical practice and were selected based on the odor prism hypothesis [36,37,38], the pioneer study by Hummel et al. [20], and specific biological mechanisms of EO constituents that facilitate the recovery of the olfactory sense by suppressing inflammation and enhancing regeneration [36].

This study aimed to give a wide characterization of rose (*R. damascena* flower oil, EO1), eucalyptus (*E. globulus* leaf/twig oil, EO2), lemon (*C. limon* peel oil, EO3), and clove (*E. caryophyllus* stem oil, EO4) EOs included in a commercial kit (StimuScent^®^) for OT, through the conjunct evaluation of their chemical composition, sensory profile in healthy participants and in subjects with hyposmia, and effects on viability/morphology in cancer and normal cells. Moreover, the EOs’ sensory properties/bioactivities were related to the main in silico physicochemical properties of their most abundant volatile constituents.

The main volatile component of the commercial product EO1 was isopropyl hexadecanoate (96.4%), an odorless organic compound known to function as an emollient, moisturizer, and thickening agent, used for the dilution of rose EO (approximately 1:20 *w*:*w*) due to its high cost of production [39], as indicated by the supplier.

Excluding isopropyl hexadecanoate, GC-MS analysis revealed citronellol (43.2%) as the major component of rose EO1, followed by phenyl ethyl alcohol, geraniol, and citronellol derivatives. A previous study reported citronellol as the major compound (35.23%) in rose EO, followed by geraniol (22.19%), nonadecane (13.85%), and nerol (10.26%) [40]. Other studies have shown that citronellol (15.9–35.3%) and geraniol (8.3–30.2%) are the major chemical constituents in rose EO, followed by lower amount of nerol (4.0–9.6%), nonadecane (4.5–16.0%), heneicosane (2.6–7.9%), and phenyl ethyl alcohol (0.6–2.9%), evidencing large differences in rose EO composition concerning the geological provenience [36].

The monoterpene cyclic ether 1,8-cineole (83%), para-cymene, and α-pinene represented the most abundant volatile compounds found in eucalyptus EO2. 1,8-Cineole (eucalyptol) has been amply reported in the literature as the major constituent (60–90%) of *E. globulus* Labill EO [1,2,36,41]. According to our results, a previous study individuated, by GC/MS analysis, 1,8-cineole (63.1%), p-cymene (7.7%), α-pinene (7.3%), and limonene (6.9%) as the main components of EO obtained by steam distillation of *E. globulus* fresh leaves [41].

GC-MS analysis allowed us to identify limonene (55.9%), β-pinene, and γ-terpinene as the most abundant components in lemon EO3, followed by small amounts of α-pinene, sabinene, para-cymene, geranial, and myrcene. Our findings on EO3 composition were perfectly in line with previous literature data that indicated limonene as the major compound of lemon peel EO [2,10,42,43,44]. Limonene (59.64%) has been indicated as the main constituent of EO obtained by hydrodistillation from *C. limon* peel, followed by γ-terpinene (19.03%), β-pinene (5.76%), β-bisabolene (1.41%), and α-bergamotene (0.84%) [44].

Our results showed that clove EO4 was characterized by high amounts of eugenol (86.6%), followed by lower amounts of (*E*)-caryophyllene and α-humulene. The terpenic compound eugenol generally represents the most prominent chemical constituent of clove EO [36,45]. A previous study reported eugenol (82.7%), followed by eugenyl acetate (15.6%) and caryophyllene (0.85%), as the main components in clove EO obtained via steam distillation [45].

Then, the odor dimensions (pleasantness, intensity, and familiarity) of selected EOs were assessed in healthy participants and participants with hyposmia using a 7-points labeled hedonic Likert-type scale, a method previously used for the evaluation of the aroma perception of flavored marine salt and an aromatic liqueur [21,22].

In healthy participants, the odor of EO3 (lemon) emerged as the most pleasant, intense, and familiar among the four tested EOs. The odor of EO4 (clove) was perceived as the least pleasant, whereas EO1 (rose) odor was indicated as the least intense, partly due to dilution. In participants with hyposmia, EO1 odor emerged as the most pleasant, while EO4 odor was perceived as the most intense.

Despite dilution, the rose scent of EO1 was recognized by both groups of participants, mainly attributable to citronellol (fresh rosy/rose odor), phenyl ethyl alcohol (rose-like, honey, spice, and lilac odor), and geraniol (sweet rose, floral, and geranium odor) [27,46].

Several participants of both groups recognized eucalyptus (EO2). However, numerous odor perceived attributes were indicated by healthy participants and those with hyposmia due to the characteristic fresh minty, woody, and camphoraceous aroma of this EO, mostly attributable to the main volatile components 1,8-cineole (camphor-like odor), ortho-cymene (sweet and citrus-like), and α-pinene (turpentine/pine odor) [27].

The lemon/citrus scent was recognized for EO3 by both groups of participants, essentially attributable to the main component limonene (citrus-like odor) [27]. Other volatiles like β-pinene (turpentine, dry, woody, and resinous odor), γ-terpinene (herbaceous-citrusy odor), α-pinene (turpentine and pine odor), and sabinene (warm, oily-peppery, and woody-herbaceous) [27] could probably contribute to the other olfactory notes/subjective odor attributes indicated by both groups.

Both healthy participants and those with hyposmia recognized the clove-like aroma of EO4. The other EO4 odor descriptors indicated by both groups were related to the complex sensory properties of EO4 main components including eugenol (cloves, warm, spicy, and floral odor), (*E*)-caryophyllene (woody-spicy, dry, and clove-like aroma odor), and α-humulene (woody, oceanic-watery, and spicy-clove odor) [27].

In both groups of participants, a positive correlation was found between the odor pleasantness of EO1, EO2, EO3, and EO4 and their odor familiarity, indicating that familiarity with an odor stimulus increases its perceived pleasantness and positive evaluation. A previous study conducted on the odor perceptions of several EOs used in aromatherapy with emotion regulation functions evidenced a positive correlation between odor pleasantness and familiarity and a weak correlation between subjective odor intensity and emotional perception, suggesting that users’ cultural characteristics are important factors that affect the EO odor perception in aromatherapy [16].

The beneficial effects of EOs in aromatherapy result from inhaling the EOs’ volatile elements. After inhalation, EOs’ volatile components typically act through the olfactory and respiratory pathways [47,48]. Inhalation aromatherapy consists of olfactory stimulation principally through the binding of odorant molecules to olfactory receptors located in olfactory sensory neurons and the activation of the olfactory nerve extending from the nose toward the brain [17,47,48]. Several chemical and physical properties of volatile/odorant molecules (including molecular size, volatility, hydrophobicity, and the presence of specific functional groups) influence their interaction with olfactory receptors, ultimately determining the perceived odor [47,48]. After inhalation, some highly volatile molecules can directly enter the brain and regulate the neuronal pathways, bypassing the entire olfactory signaling [48]. In the olfactory pathway, three modes of EO cell-to-cell diffusion exist, including the transcellular, paracellular, and intracellular pathways, with the help of the olfactory nerve and olfactory bulb [48]. EOs’ therapeutic effects are due to their close structural resemblance to the physiological neurotransmitters and hormones [47]. The respiratory pathway involves the entry of EOs into the brain region through the respiratory cerebellum, and the trigeminal innervations also provide a strong link between the nose and the brain drug-delivery system [48].

A positive correlation (r = 0.7314) was evidenced in healthy participants, but not in those with hyposmia, between the EO’s odor intensity and vapor pressure (VP) values of their main components (citronellol, 1,8-cineole, limonene, and eugenol), indicating that EO compound volatility partly affected the intensity of olfactory perception [47,48]. Participants with hyposmia perceived EO4 odor as the most intense among the tested EOs, probably due to the ability of eugenol to stimulate the trigeminal nerve [49]. The stimulation of the trigeminal nerve can improve olfactory function in individuals with hyposmia, potentially enhancing the ability to perceive olfactory information [50].

*R. damascena* EO (representing flowery scent), obtained from the flower petals, has been reported to possess anti-inflammatory, antiviral, trachea relaxant, and immunological activity [17,36,39]. Moreover, several activities of rose EO on CNS have been reported, including stimulatory GABAergic, antidepressant, relaxing, sedative, analgesic, and anxiolytic properties [17]. The major chemical constituents in rose EO, citronellol and geraniol, have been proposed as responsible for EO pharmacological activities, such as antidepressant, anti-dementia, hypoglycemic, anti-inflammatory, analgesic, antioxidant, anticonvulsant, antimicrobial, and anti-SARS-CoV-2 effects [5,36,39].

Several activities have been reported for eucalyptus (*E. globulus*) EO (herbal/menthol, resinous scent), obtained from leaves, including anti-inflammatory (in bronchial asthma, respiratory diseases, and rhinosinusitis), antioxidant, immunostimulant, and anti-anxiety properties [17,36,41]. Eucalyptus EO components 1,8-cineole, α-pinene, and p-cymene are well known for their anti-inflammatory effects [36].

Several studies highlighted the anti-inflammatory (in asthma, chronic bronchitis, and allergic rhinitis), neuroimmunomodulatory, immunostimulating, antimicrobial, anticancer, antidepressant, mentally stimulating, anxiolytic, analgesic, and positive mood reviving activity of lemon EO (citrus/fruity scent) obtained from the fruit peel/pericarp [17,36]. Limonene, the main chemical constituent of lemon peel EO, has been indicated as the principal responsible for the anticancer, anti-inflammatory, and antimicrobial properties of the extract [2,3,7,10,42,51].

Clove EO (spicy/aromatic scent), isolated from flower buds, demonstrated anti-inflammatory, antiviral, anticancer, immunomodulatory, antioxidant, and antimicrobial activity [17,36,45,52]. The terpenic compound eugenol, the most prominent chemical constituent of clove EO, is known to have anticancer, anti-inflammatory, antioxidant, and antimicrobial effects [36,45,52,53].

Some EO molecules can pass through the olfactory mucosa (olfactory pathways), depending on their molecular sizes. The EO’s penetration through the olfactory nerve, which is connected to brain areas, allows for the generation of several cellular and molecular events (regulation of neuronal pathways) [48]. Therefore, we explored the impact of selected EOs in cell systems, monitoring their ability to affect cell viability and morphology, and induce apoptosis/necrosis in SH-SY5Y human neuroblastoma cells, a cell line extensively used in neuroscience research as an in vitro neuronal cell model [23,24].

In our experimental conditions, EO3 emerged as the most cytotoxic oil against cancer SH-SY5Y cells (IC_50_ value = 77.5 μg/mL after 24 h of incubation), and the order of potency was EO3 > EO4 > EO2 > EO1. After 1 day of incubation, EO3 significantly increased the number of apoptotic cells (rounded morphology). No viable cells were observed by phase contrast microscopy after a long treatment (6 days) with EO3 at 50 μg/mL, suggesting a noteworthy anticancer effect on neuroblastoma cells. EO3 exhibited a more marked toxic effect in cancer cells than in human skin HaCaT fibroblasts, evidencing certain selectivity versus tumor cells.

Several investigations have shown the growth inhibitory effect and antiproliferative properties of *C. limon* EO in various cancer cells (HeLa, PC-3, A549, and MCF-7 cell lines) [51]. We previously demonstrated the cytotoxic effect (24 h of incubation, MTT assay) of the EO obtained from the peel of *Citrus limon* var. *pompia* in B16F10 melanoma cells (IC_50_ value = 148 μg/mL) and cancer HeLa cells (IC_50_ value = 408 μg/mL) [10]. The cytotoxicity of lemon peel EO has been largely ascribed to its main component limonene, which showed cytotoxicity and pro-apoptotic effect against various cancer cell lines [7,10,42,51]. Moreover, limonene activated autophagy in SH-SY5Y human neuroblastoma and MCF7 human breast cancer cell lines [11].

After 24 h of incubation, EO4 was less toxic than EO3 in neuroblastoma SH-SY5Y cells; however, it still exerted a significant toxic effect (IC_50_ value = 120.5 μg/mL), affecting cancer cell viability and morphology. The presence of apoptotic cells and cells with a more fusiform shape and increased processes was observed after 24-incubation in SH-SY5Y cells, suggesting a potential effect of clove EO4 in inducing their differentiation into a neuronal phenotype [23,24]. EO4 showed a lower toxic effect in HaCaT keratinocytes (IC_50_ value = 255.5 μg/mL after 24 h incubation), highlighting a more selective toxicity towards malignant cells. A significant increase in the number of apoptotic cells was also evidenced after a long treatment (6 days) with EO4 at 50 μg/mL, evaluated by PI fluorescence assay (red fluorescence), which evidences late apoptotic and necrotic cells [23,24]. The presence of longer and more intense neurites (processes) (increase in NF green fluorescence) was also observed after 6 days of treatment with EO4, highlighting a potential effect on cell differentiation to a neuronal phenotype.

Previous studies evidenced the antiproliferative activity of clove EO against cervical (HeLa), pancreatic (Panc), colon (HCT), liver (HepG2), breast (MCF-7), prostate (PC3), colon (HCT116), thyroid (HTh-7), and lung (A549) cancer cells [52,53]. Clove EO showed a selective cytotoxic activity against melanoma (RPMI-7951) and colorectal adenocarcinoma cells (HT-29) after 48 h of incubation, without significantly affecting normal cells, and its cytotoxic activity was mainly attributed to its high eugenol content [52].

EO2 (eucalyptus) exhibited a cytotoxic effect in SH-SY5Y cells after 24 h of incubation at the dose range of 100–250 μg/mL, inducing an increase in the number of cells with an apoptotic (rounded) morphology. No changes in cell viability/morphology were observed in HaCaT skin keratinocytes after 24 h of incubation, indicating a selective toxicity towards malignant cells. A significant increase in the number of apoptotic/necrotic cells was also observed after six days of treatment in the presence of EO2 at a dose of 50 μg/mL, coupled with a slight reduction in cell viability.

*E. globulus* EO has been found to decrease the viability of human colon cancer cells SW48 in concentrations of 0.05%, 0.1%, 0.5%, and 5% after 48 h of incubation; however, concentrations of 0.5% and 5% also proved to be significantly toxic in normal fibroblast cells [54]. This EO exhibited toxic effects on HEK293t and HEPG2 cell lines at high dose (0.5%), with IC_50_ = 0.2% for both cell lines [54]. Moreover, eucalyptus EO showed the ability to induce apoptosis in tumor cells [7]. Previous studies demonstrated the ability of the monoterpene 1,8-cineole/eucalyptol to induce apoptosis in human colon cancer cell lines HCT116 and RKO, and this effect was associated with the inactivation of surviving and activation of p38 [9].

The incubation for 24 h with EO1 induced a significant viability reduction and a decrease in the total cell number in cancer SH-SY5Y cells at the dose of 250 μg/mL, without effect on cell morphology. EO1 did not induce marked changes in cell viability/morphology in HaCaT skin keratinocytes (24 h of incubation), indicating a more selective toxicity towards malignant cells than normal cells. Moreover, a significant slight reduction in cell viability was observed after six days of treatment in the presence of EO1 at the dose of 50 μg/mL, coupled with a significant increase in the number of apoptotic cells (IP-stained rounded cells). It should be underlined that the biological effects observed in EO1 did not accurately represent the activity of authentic rose oil, due to the presence of isopropyl hexadecanoate as diluent. However, EO1 exhibited interesting properties despite the high dilution of rose EO.

Several studies have shown the anticancer activity of rose EO and its potential to be used as adjuncts in adjuvant therapy of tumors [55,56,57]. Rose EO has been demonstrated to possess cytotoxic and genotoxic activity, clearly dependent on the concentration applied and the cell type treated [55,56,57]. A previous study evidenced that the rose EO was safe at a dose <50 μg/mL when applied to normal human blood lymphocytes (by 7-AAD assay and DNA index determination), while it exerted antitumor activity against human HepG2 and MCF7 cancer cells with IC_50_ values of 13.03 ± 0.8 and 16.44 ± 1.4 μg/mL, respectively [55]. Another study evidenced that the EO of *R. damascena* Mill. from Iran was safe at a low dose (10 μg/mL) both in normal NIH3T3 fibroblasts and A549 cancer cells (48 h of incubation), but an increase in cytotoxic and genotoxic effects was observed at the dose range of 50–200 μg/mL (assessed by micronucleus assay), with an higher toxicity in cancer (IC_50_ = 6.43 ± 3.373 μg/mL) than in normal NIH3T3 cells (IC_50_ = 42.93 ± 0.502 μg/mL) [56]. The main volatiles citronellol and geraniol demonstrated inhibitory activity on cell growth in various cancer cell lines [55,56]. Moreover, rose EO and citronellol triggered apoptosis in human lung A549 cancer cells [57].

Numerous studies have highlighted the beneficial pharmacological effects of rose, eucalyptus, lemon, and clove EOs after inhalation on the CNS, and the respiratory and olfactory pathways are probably involved in their action in the human body system [48]. Our data showed that these EOs, amply used in OT, affected viability and morphology in a neuronal cell model (SH-SY5Y cells) [23,24], with less effect in normal skin fibroblasts.

The anticancer activity of small molecules depends on their ability to penetrate biological membranes (bioavailability) and modulate intracellular signaling pathways and molecular targets [24,58]. A Log P between 1 and 3 (lipophilicity) has been indicated as favorable for oral bioavailability (good permeability and solubility) [24,32,58]. The anticancer activity of EOs has been associated with their ability to cross/permeabilize the cell membrane and coagulate the cytoplasm, thus damaging lipids and proteins [2,4,10,58]. Due to their lipophilic nature, EO volatile components are able to easily penetrate biological membranes, induce alterations in cell membrane fluidity, and stimulate mitochondrial membrane depolarization, thus leading to necrosis and apoptosis [2,4,10,58].

The Bioavailability radars of the main EO components citronellol, 1,8-cineole, limonene, and eugenol, qualified them as orally bioavailable, mostly entering into the pink area [28,33]. All compounds possess the capability to cross cell membranes as indicated by the percentages of compounds predicted to be absorbed through the human intestine (% HIA ranging from 92.041 to 96.505%) [29,32]. In our experimental condition, EO3 emerged as the most toxic in cancer cells, but also in normal cells to a lesser extent. Its principal component limonene exhibited the lowest values of MW (136.23) and TPSA (0 Å), and the highest hydrophobicity (values of 3.4 and 3.27 for XLogP3-AA and Consensus Log Po/w, respectively), among other main volatiles. Limonene is a highly volatile lipophilic aroma compound, and previous studies highlighted its ability in cancer cells to cross biological membranes, induce alterations in cell membrane fluidity, enhance the intracellular ROS generation, stimulate mitochondrial membranes depolarization, thus leading to apoptosis and necrosis [1,7,9,10].

Apart from olfactory stimulation, another prominent pathway taken by the EOs to affect brain functioning is through their alveolar absorption after inhalation [48]. Alveolar diffusion enables the EO molecules to reach the systemic circulation, cross the BBB, and thereby potentially interact with specific CNS regions. Molecular parameters, such as MW, TPSA, and lipophilicity, have been reported to influence the permeability/diffusion of volatile molecules across the BBB [23,24,59]. Molecules with higher Log *p* values have been proposed to penetrate better through the BBB, and for CNS drugs, a high Log P range (2–5) may be optimal to cross the BBB [23,24,29]. In the pkCSM-pharmacokinetics model, molecules with a log BB < −1 are considered poorly distributed to the brain, while compounds with values > 0.3 readily cross the BBB [24,29,34]. A great BBB permeability (by passive diffusion) was predicted for citronellol, 1,8-cineole, limonene, and eugenol by the Swiss-ADME (BOILED Egg model) [28,33]. Moreover, values of 0.627, 0.368, 0.732, and 0.374 log BB were computed for citronellol, 1,8-cineole, limonene, and eugenol, respectively, by pkCSM-pharmacokinetics, confirming their ability to cross the BBB. In addition, compounds with a log PS > −2 (CNS permeability–distribution by pkCSM-pharmacokinetics) are considered to penetrate the CNS, while those with log PS < −3 are considered unable to penetrate the CNS [24,29,34]. CNS permeability values of −2.222, −2.972, −2.37, and −2.007 log PS [29] were computed for citronellol, 1,8-cineole, limonene, and eugenol, respectively, highlighting, for tested EOs, the potential ability to cross the BBB and penetrate the CNS after inhalation, and to exert an in vivo anticancer activity.

Limitations of this study include the low number of participants with hyposmia for EO sensory assessment, and the partial biological effects observed in EO1, due to the presence of the diluent isopropyl palmitate.

## 4. Materials and Methods

### 4.1. Materials

Chemicals for cell culture, including penicillin, streptomycin, fetal bovine serum (FBS), Dulbecco’s Modified Eagle’s Medium (DMEM), and Trypsin 0.25%-EDTA, were acquired from EuroClone (Pero, MI, Italy). Dimethyl sulfoxide (DMSO) and 3-(4,5-dimethylthiazol-2-yl)-2,5-diphenyltetrazolium bromide (MTT) were purchased from Merck Life Science (Milan, Italy). Propidium iodide (PI) was acquired from Thermo Fisher Scientific (Waltham, MA, USA).

### 4.2. EOs Used for the Study

StimuScent Dos Medical smell training-SET 1 (Lot: SO-29082022-01) with EOs of rose (*R. damascena* flower extract, EO1), eucalyptus (*E. globulus* leaf/twig oil, EO2), lemon (*C. limon* peel oil, EO3), and clove (*E. caryophyllus* stem oil, EO4) were purchased from Dos Medical (Sense Trading BV, Groningen, The Netherlands).

### 4.3. Analysis of EOs Composition by GC-MS

EOs analysis was carried out by gas chromatography/mass spectrometry (GC-MS), using a gas chromatograph (Agilent 7820A, Agilent Technologies, Santa Clara, CA, USA) equipped with an HP-5MS UI capillary column (30 m × 0.25 mm i.d., with 0.25 µm stationary film thickness) (Agilent J&W, Agilent Technologies, Santa Clara, CA, USA) coupled with a mass selective detector having an electron ionization device (EI) and a quadrupole analyzer (Agilent 5975, Agilent Technologies, Santa Clara, CA, USA), as previously reported [26]. The following temperature program was used: from 60 °C to 250 °C at a rate of 3 °C min^−1^ and then held at 250 °C for 20 min (total analysis time 83 min). Other operating conditions were the following: carrier gas helium (purity ≥ 99.9999 %, Air Liquide, Assemini, CA, Italy); flow rate, 1.0 mL/min; injector temperature, 250 °C; detector temperature, 300 °C. Injection of 1 μL of diluted sample (1:100 in n-hexane, *w*/*w*) was performed with a 1:10 split ratio, using an autosampler (Agilent Model 7683, Agilent Technologies, Santa Clara, CA, USA).

The MS conditions were as follows: MS transfer line temperature 240 °C; EI ion source temperature, 200 °C with ionization energy of 70 eV; quadrupole temperature 150 °C; scan rate, 3.2 scan s-1 at *m*/*z* scan range, (30 to 480). The software MSD ChemStation G1701EA (rev. E.01.00.237, Agilent Technologies, Santa Clara, CA, USA) was used to handle and process chromatograms and mass spectra. Compounds were identified by comparison of their mass spectra with those of libraries spectra, Adams [30] and NIST02 [31]. The results were further confirmed by comparison experimental retention indices with their reported in the literature [30]. Retention indices of the components were determined relative to the retention times of a series of n-alkanes (two standard mixes C_8_–C_20_ and C_21_–C_40_) with linear interpolation [60]. Semi-quantitative analysis was performed by peak area normalization, and the number of individual components was expressed as % peak area, as previously reported [26,61].

### 4.4. Participants to EOs Sensory Analysis

A total population of 30 participants (16 women and 14 men) was enrolled. All participants involved in the study received an explanatory statement and signed a written informed consent. The participants did not take any medications for 5 days before the test. Exclusion criteria were neurodegenerative disorders, psychiatric conditions, stroke, and head/neck injury, as previously reported [21,22]. Data collections were performed from November 2024 to February 2024. Demographic features (age, weight, height, and body mass index) were collected for all participants. The Ethical Committee of the University of Cagliari (Protocol number: 3605 10/01/24) approved this study, performed according to the Declaration of Helsinki.

### 4.5. Evaluation of Olfactory Functions of Participants

The assessment of olfactory function was performed with the Sniffin’ Sticks test (Burghart Messtechnik, Wedel, Germany) (Figure 1a), a validated and commonly used tool for routine clinical assessment of olfactory function in subjects with a normal sense of smell and in individuals with smell loss [21,22]. This test consists of pen-like odor-dispensing devices for the evaluation of odor threshold (OThr), odor discrimination (ODi), and identification (OId) as previously reported [21,22]. No participants were allowed to smoke or use scented products before the chemosensory evaluation. The OThr scores were assessed using 16 stepwise dilutions of n-butanol and ranged from 16 (perception of the lowest n-butanol concentration) to 1 (no perception of the highest concentration). For the ODi test, three different pens were used, two containing the same odor and the third containing the odor target and the ODi scores ranged from 0 to 16 (sum of correct answers). The OId test was assessed by 16 common odors as previously reported. The sum of OThr + ODi + OId represented the total olfactory score (TDI), and values ≤ 16, between 16.25 and 30.5, between 30.75 and 41.25, and >41.5, indicated functional anosmia, hyposmia, normosmia, and super smellers, respectively [21,22].

### 4.6. Assessment of EOs Odor Pleasantness, Intensity, and Familiarity

Non-trained subjects (30 participants), 22 healthy controls and 8 patients with hyposmia) were enrolled to assess the sensory properties of EO1, EO2, EO3, and EO4 (Figure 1b). Before the sensory assessment, each EO was aliquoted at room temperature (23 °C) in 2 mL glass test bottles. The odor pleasantness, intensity, and familiarity of EOs were evaluated using a self-reported 7-point Likert-type scale (a hedonic scale method), which ranged from 0—not at all to 6 (such as 0 = very unpleasant and 6 = very pleasant; 0 = not intense at all and 6 = very intense; 0 = not familiar at all and 6 = very familiar) [21,22]. A value of 3 was considered a neutral point. The labeled hedonic Likert Scale is amply used in food science to measure hedonic differences among beverages, foods, and consumer products and predict their acceptance, suitable for use without extensive training [21,22]. Moreover, the subjects generated subjective odor (aroma) attributes/descriptors perceived with more intensity.

### 4.7. Cell Cultures

Human neuroblastoma cell line SH-SY5Y was supplied by the Cell Bank Interlab Cell Line Collection, IRCCS San Martino Policlinico Hospital, Genova, Italy (code# HTL95013). The human HaCaT keratinocyte cell line, spontaneously immortalized cells derived from normal human skin cells, was obtained from CLS-Cell Line Services (Eppelheim, Germany). Cells were grown in a DMEM high glucose medium enriched with 10% *v*/*v* FBS and 100 units/mL of streptomycin/penicillin in a humidified atmosphere of 5% CO_2_ at 37 °C. The cells were propagated by changing the medium every two days and split weekly when confluent using a Trypsin 0.25%-EDTA solution.

### 4.8. EOs Effects on Cell Viability After 24 h of Incubation (MTT Assay)

The cytotoxic effect of the four EOs was measured in cancer SH-SY5Y cells and HaCaT cells by the MTT viability colorimetric assay, as previously reported [24,26]. The effect on cell viability of different amounts of DMSO (vehicle used to dissolve the compounds) was also evaluated. SH-SY5Y neuroblastoma and HaCaT cells were seeded (at a density of 10^5^ cells/mL) in 96-well plates in a complete culture medium (100 μL). After 48 h-incubation, SH-SY5Y cells were treated with EO1, EO2, EO3, and EO4 (5–500 μg/mL, from a 50 mg/mL solution in DMSO) or DMSO (from 0.01 to 1% *v*/*v*) in a complete fresh medium for 24 h, whereas control cells (untreated-cells) were cultured for 24 h in a complete fresh medium. Then, after medium removal, control (non-treated) cells, compound-treated cells, and cells incubated with DMSO (vehicle-treated cells) were subjected to the MTT assay as previously reported [24,26]. The auto microplate reader (Infinite 200, Tecan, Grödig, Austria) was used to measure color development at the wavelength of 570 nm. Results of cell absorbance, proportional to the number of viable cells, were expressed as a percentage of cell viability compared to control (untreated) cells (100% viability).

In another set of experiments, SH-SY5Y neuroblastoma cells were seeded (at a density of 10^5^ cells/mL) in 96-well plates and cultured for 6 days in culture medium (100 μL) containing 50 μg/mL of each EO or DMSO (0.1% *v*/*v*), whereas control cells (untreated-cells) were cultured for the same time only in culture medium. Then, after medium removal, cells were subjected to the MTT assay as described above.

Morphological observations of control SH-SY5Y and HeLa cells and cells after 24 h or 6 days of incubation with various amounts of EOs and DMSO were performed by microscopic analysis with a ZOE^TM^ Fluorescent Cell Imager (Bio-Rad Laboratories, Inc., Hercules, CA, USA).

### 4.9. Propidium Iodide Apoptosis Assay in Cancer SH-SY5Y Cells

The effect of the four EOs on cell apoptosis/necrosis was assessed by staining cancer SH-SY5Y cells with propidium iodide (PI), a DNA-binding fluorescent dye (red) that enters cells with disrupted membranes and can evidence late apoptotic and necrotic cells [23,24]. SH-SY5Y neuroblastoma cells were seeded in 96-well plates (at a density of 10^5^ cells/mL) and cultured for 6 days in culture medium (100 μL) containing 50 μg/mL of each EO or DMSO (0.1% *v*/*v*), whereas control cells (untreated cells) were cultured for the same time only in culture medium. The concentration of 50 μg/mL was selected based on preliminary data on SH-SY5Y cell viability/morphology after 24 h of incubation with EOs. After incubation and medium removal, SH-SY5Y cells were incubated with PI (final concentration 1 μg/mL), according to the guidelines provided by the manufacturer. After dark incubation with PI (2 h), microscopic observations were made using a ZOE^TM^ Fluorescent Cell Imager. The offset values and instrument gain were adjusted using control cells. ImageJ software (version 1.53e) was used for the evaluation of PI images (six different images were processed for each sample). Background fluorescence was subtracted from images and fluorescence intensity of treated cells was expressed as % of control cell fluorescence.

### 4.10. Analysis of Neurofilaments by Immunofluorescence in Cancer SH-SY5Y Cells

SH-SY5Y neuroblastoma cells were cultured for 6 days in culture medium (100 μL) containing 50 μg/mL of each EO or DMSO (0.1% *v*/*v*), whereas control cells (untreated cells) were cultured for the same time only in culture medium. The concentration of 50 μg/mL was selected based on preliminary data on SH-SY5Y cell viability/morphology after 24 h of incubation with EOs. After incubation and medium removal, the immunofluorescence demonstration of neurofilaments was performed on methanol-fixed SH-SY5Y neuroblastoma cells according to literature [23,62]. After rehydration in phosphate-buffered saline (PBS) with 0.2% Triton-X-100, the cells were incubated with 20% normal goat serum in PBS for 30 min. A mouse monoclonal antibody to neurofilaments (NF) (N2912 Sigma-Aldrich, Burlington, MA, USA) was used as a primary antiserum. Alexa Fluor 488-conjugated polyclonal goat anti-mouse IgG antibodies (A-11001, Invitrogen, Waltham, MA, USA) were the secondary antisera. Cells were washed with PBS-Triton after each step. The nuclei were detected by counterstaining with Hoechst-33342. In negative control cells, the specificity of the antisera was evaluated by substituting the primary antibody with normal serum. Microscopic observations were made using a ZOE^TM^ Fluorescent Cell Imager.

### 4.11. Physicochemical and Pharmacokinetic Properties of EOs Main Components by in Silico Evaluation

Physicochemical properties (MW, XLogP3-AA, HBDC, HBAC, RBC, TPSA, and complexity) and simplified molecular-input line entry specification (canonical SMILES) nomenclature of the main EOs components, including isopropyl hexadecanoate, 1,8-cineole, limonene, β-pinene, eugenol, and (*E*)-caryophyllene, were obtained from the free web database PubChem [27]. The canonical SMILES of the compounds were introduced into the freely accessible web tools SwissADME [28] and pkCSM-pharmacokinetics [29]. The SwissADME web tool, which provides a global evaluation of the pharmacokinetics profile of small molecules, was used to estimate compound lipophilicity (Consensus Log P_o/w_), water solubility (Log S), gastrointestinal absorption (according to the white of BOILED-Egg model), and BBB permeation (according to the yolk of the BOILED-Egg model) [28,32,33]. The pkCSM-pharmacokinetics, a method for predicting and optimizing small-molecule pharmacokinetics/toxicity properties, was employed to calculate compound intestinal absorption (IA, %), the logarithm of the ratio of compound concentration in the brain and the blood (log BB), and the logarithm of blood-brain permeability-surface area product (log PS) [29,34].

### 4.12. Statistical Analyses

GraphPad Prism version 10.0.0 for Windows (GraphPad Software, Boston, MA, USA) was used to estimate the statistical differences between different data groups. Multiple comparisons of the group means were evaluated by One-way analysis of variance (One-way ANOVA) followed by the Bonferroni Multiple Comparisons Test. Student’s unpaired *t*-test with Welch’s correction, which does not require the assumption of equal variance between populations, was used to compare the means of two data groups. The minimal level of significance was *p* < 0.05. Bivariate correlations using Pearson’s coefficient (r) were performed to evaluate potential correlations between EOs odor dimensions.

## 5. Conclusions

Numerous studies have highlighted the beneficial pharmacological effects of EOs in-halation on the CNS and the OT role in improving the sense of smell in patients with olfactory deficits. In this study, we evaluate the chemical composition, sensory perception in non-trained subjects, and bioactivity in cell models of rose, eucalyptus, lemon, and clove EOs, amply used in OT. Citronellol, 1,8-cineole, limonene, and eugenol emerged as the most abundant volatile compounds in rose, eucalyptus, lemon, and clove EOs, respectively. Our data evidenced some differences in the perception of EOs odor dimensions (pleasantness, intensity, and familiarity) in healthy participants and subjects with hyposmia. Lemon EO emerged as the most pleasant, intense, and familiar in healthy participants, whereas rose and clove EOs emerged as the most pleasant and intense, respectively, in participants with hyposmia. Interestingly, the odor intensity perception of EOs appeared to be related to the volatility of their main volatile constituent in healthy subjects, whereas the stimulation of the respiratory pathway (trigeminal nerve) was probably preeminent in EO odor intensity perception in participants with hyposmia. We also demonstrated the ability of the four EOs to affect cell growth/morphology in human neuroblastoma SH-SY5Y cells, with a lesser effect shown in normal skin fibroblasts. Taking into consideration the high lipophilicity and the computed ability to cross the BBB and permeate the CNS of main volatiles, the potential in vivo anticancer effects and a certain cell toxicity of rose, eucalyptus, lemon, and clove EOs after inhalation could not be excluded.

Taken together, our results provide new insights into the sensory perception, bioactivity, and relationship between the composition–activity of rose, eucalyptus, lemon, and clove EOs, useful for a more targeted OT application.

## Figures and Tables

**Table 1:** Tentative identification of volatile compounds of EO1, EO2, EO3, and EO4. The results were evaluated by GC-MS and semi-quantitative analysis was performed by peak area normalization (expressed as % peak area). Retention time (Tr), retention time retention index (RI_EXP_), retention index from literature (RI_LIT_), formula, and class of identified compounds were also reported.

						EO1	EO2	EO3	EO4
Compound	Tr	^1^ RI_EXP_	RI_LIT_	Formula	Class	% Peak Area			
α-Thujene	5.26	927	924	C_10_H_16_	MH	-	-	0.5	-
α-Pinene	5.47	935	932	C_10_H_16_	MH	-	4.9	2.9	-
Camphene	5.91	951	946	C_10_H_16_	MH	-	-	0.1	-
Sabinene	6.52	973	969	C_10_H_16_	MH	-	-	2.8	-
β-Pinene	6.70	980	974	C_10_H_16_	MH	-	0.3	15.5	-
Myrcene	6.96	989	988	C_10_H_16_	MH	0.1	0.7	1.6	-
α-Phellandrene	7.52	1007	1002	C_10_H_16_	MH	-	1.0	-	-
α-Terpinene	7.88	1017	1014	C_10_H_16_	MH	-	0.2	0.1	-
para-Cymene	8.15	1024	1020	C_10_H_14_	MH	-	7.9	2.3	-
Limonene	8.41	1031	1024	C_10_H_16_	MH	-	-	55.9	-
1,8-Cineole	8.51	1034	1026	C_10_H_18_O	MO	-	82.7	-	-
(E)-β-Ocimene	8.86	1044	1044	C_10_H_16_	MH	-	-	0.1	-
γ-Terpinene	9.33	1057	1054	C_10_H_16_	MH	-	2.3	11.1	-
Terpinolene	10.31	1083	1086	C_10_H_16_	MH	-	-	0.4	-
Phenyl ethyl alcohol	11.29	1109	1106	C_8_H_10_O	Other	1.4	-	-	-
Dihydro citronellol	14.77	1192	1194	C_10_H_22_O	MO	0.3	-	-	-
Citronellol	16.11	1224	1223	C_10_H_20_O	MO	2.3	-	-	-
Neral	16.55	1234	1235	C_10_H_16_O	MO	-	-	1.1	-
Geraniol	17.09	1246	1246	C_10_H_18_O	MO	0.8	-	-	-
Geranial	17.84	1263	1264	C_10_H_16_O	MO	-	-	2.1	-
Citronellyl acetate	21.36	1346	1350	C_12_H_22_O	MO	0.1	-	-	-
Eugenol	21.65	1353	1356	C_10_H_12_O	MO	-	-	-	86.6
(*E*)-Caryophyllene	24.18	1412	1418	C_15_H_24_	SH	-	-	0.2	10.2
Linalol butanoate	24.29	1415	1421	C_14_H_24_O	MO	0.1	-	-	-
α-*trans*-Bergamotene	24.79	1427	1432	C_15_H_24_	SH	-	-	0.6	-
α-Humulene	25.64	1448	1452	C_15_H_24_	SH	-	-	-	2.5
β-Bisabolene	27.83	1502	1505	C_15_H_24_	SH	-	-	0.8	-
δ-Amorfene	28.19	1511	1511	C_15_H_24_	SH	-	-	-	0.2
*trans*-Calamelene	28.30	1513	1521	C_15_H_22_	SH	-	-	-	0.1
(*Z*)-Dihydro-apofarnesol	30.60	1572	1572	C_14_H_26_O	MO	-	-	-	0.4
Isopropyl hexadecanoate	46.42	2026	2024	C_19_H_38_O_2_	Other	94.8	-	-	-
Unidentified peaks						0.1	-	2.0	-

^1^ RI_EXP_, retention index determined on a HP-5MS fused silica column relative to a series of n-alkanes. Compounds were identified by comparing their retention indices (RI_EXP_) and mass spectra (MS) with those reported in Adams (RI_LIT_) [30] and NIST02 [31] libraries. MH, represent monoterpene hydrocarbons; MO, oxygen-containing monoterpenes; SH, sesquiterpene hydrocarbons.

**Table 2:** Demographic and clinical features of participants with hyposmia (Hyp) compared to healthy participants (Ctrl).

Parameters	Ctrl (n = 22)	Hyp (n = 8)
Age	37.8 ± 16.0	50.8 ± 14.4
Sex	13 W/9 M	3 W/5 M
Weight (kg)	61.9 ± 10.0	67.0 ± 9.6
Height (m)	1.6 ± 0.1	1.7 ± 0.1 *
BMI	23.4 ± 3.7	23.7 ± 1.7
OThr	8.4 ± 2.5	2.3 ± 1.5 ***
ODi	13.1 ± 1.7	10.3 ± 1.2 ***
OId	13.5 ± 1.6	10.2 ± 3.0 ***
TDI score	35.1 ± 3.5	22.9 ± 3.9 ***

Legend: BMI = body mass index. OThr = odor threshold; ODi = odor discrimination; OId = odor identification; TDI score = OThr + ODi + OId scores. Significant differences between groups were assessed by Student’s unpaired *t*-test with Welch’s correction. *** = *p* < 0.001; * = *p* < 0.05 versus Ctrl.

**Table 3:** Odor (aroma) perceived attributes of EO1 (rose), EO2 (eucalyptus), EO3 (lemon), and EO4 (clove) measured in healthy participants (Ctrl, n = 22) and participants with hyposmia (Hyp, n = 8). Odor descriptors of EOs’ main constituents, identified by GC-MS, from the literature data [27].

EO	EO Perceived Odor	Main Constituents	Odor [27]
EO1	Ctrl: Rose, pungent, vanilla, citrus fruits, Marseille soap, violet, flowers, Scots pine, fir, citronella. Hyp: Honey, rose, pleasant, natural essence, spice	^1^ Isopropyl hexadecanoate Citronellol Phenyl ethyl alcohol Geraniol	Odorless Fresh rosy, rose Rose-like odor Sweet rose, floral, geranium-like
EO2	Ctrl: Eucalyptus, fennel, licorice, anise, herbs, mint, menthol, balsamic plants, lavender, camphor, tiger balm, tea tree oil, peppermint. Hyp: Mild mint, unpleasant, aromatic, medicine, eucalyptus	α-Pinene para-Cymene 1,8-Cineole γ-Terpinene	Turpentine, pine Sweetish aromatic, weak citrus Camphor-like Herbaceous-citrusy
EO3	Ctrl: Lemon, Citrus, orange, lemon and orange, mandarin, ginger Hyp: Yuck, tea, orange, clementine, sweet, fresh, lemon	α-Pinene	Turpentine, pine
		Sabinene	Warm, oily-peppery, woody-herbaceous
		β-Pinene	Turpentine, dry, woody, resinous
		para-Cymene	Sweetish aromatic, weak citrus
		Limonene	Citrus-like
		γ-Terpinene	Herbaceous-citrusy
		Geranial	Lemon-like, strong lemon
EO4	Ctrl: Cloves, incense, cinnamon, mild cinnamon, smoked, spices, herbs, leather, eucalyptus. Hyp: Cloves, lavender, natural plant, unpleasant	Eugenol (*E*)-Caryophyllene α-Humulene	Cloves, warm, spicy, floral Woody-spicy, dry, clove-like aroma Woody, oceanic-watery, spicy-clove

^1^ Odorless organic compound used as a diluent.

## Data Availability

The data that support the findings of this study are available from the corresponding author upon reasonable request.

## Supplementary Materials

The following supporting information can be downloaded at: https://www.mdpi.com/article/10.3390/molecules30173591/s1, Table S1: Canonical smiles of citronellol, 1,8-cineole, limonene, and eugenol; Table S2: Physicochemical and pharmacokinetic properties of citronellol, 1,8-cineole, limonene, and eugenol; Figure S1: Full GC-MS chromatograms of EO1, EO2, EO3, and EO4. Figure S2: Bioavailability radars of citronellol, 1,8-cineole, limonene, and eugenol; Figure S3: “BOILED-Egg” graphs of citronellol, 1,8-cineole, limonene, and eugenol.
