# Supplementary material for: Chemical Characterization, Sensory Evaluation, and Biological Activity in Neuronal Cells of Essential Oils (Rose, Eucalyptus, Lemon, and Clove) Used for Olfactory Training
Source: Molecules. 2025 Sep 2;30(17):3591. doi: 10.3390/molecules30173591 (PMC12430588; doi:10.3390/molecules30173591)
Supplement: Supplementary file 1 [file molecules-30-03591-s001.zip › molecules-3805133-supplementary.pdf]

Article

Chemical Characterization, Sensory Evaluation, and Biological Activity in Neuronal Cells of Essential Oils (Rose, Eucalyptus, Lemon, and Clove) Used for Olfactory Training

Antonella Rosa [1,*], Franca Piras [1], Alessandra Piras [2], Silva Porcedda [2], Valeria Sogos [1] and Carla Masala [1]

1 Department of Biomedical Sciences, University of Cagliari, Cittadella Universitaria, SS 554, km 4.5, 09042 Monserrato, CA, Italy; fpiras@unica.it (F.P.); sogos@unica.it (V.S.); cmasala@unica.it (C.M.)
2 Department of Chemical and Geological Sciences, University of Cagliari, Cittadella Universitaria, SP 8, Monserrato-Sestu km 0.700, 09042 Monserrato, CA, Italy; apiras@unica.it (A.P.); porcedda@unica.it (S.P.)
* Correspondence: anrosa@unica.it

Table of Contents

Table S1: Canonical smiles of citronellol, 1,8-cineole, limonene, and eugenol.

Table S2: Physicochemical and pharmacokinetic properties of citronellol, 1,8-cineole, limonene, and eugenol.

Figure S1: Full GC-MS chromatograms of EO1, EO2, EO3, and EO4.

Figure S2: Bioavailability radars of citronellol, 1,8-cineole, limonene, and eugenol.

Figure S3: "BOILED-Egg" graphs of citronellol, 1,8-cineole, limonene, and eugenol.

Table S1. Canonical smiles, obtained by PubChem web database [27], of the main identified volatile components of EOs, including citronellol for EO1 1,8-cineole for EO2, limonene for EO3, and eugenol for EO4.

Compound name	[1]Canonical SMILES
Citronellol	CC(CCC=C(C)C)CCO
1,8-Cineole	CC1(C2CCC(O1)(CC2)C)C
Limonene	CC1=CCC(CC1)C(=C)C
Eugenol	COC1=C(C=CC(=C1)CC=C)O

[1]Computed by OEChem 2.3.0 (PubChem release 2025.04.14).

Table S2. Physicochemical and pharmacokinetic properties of citronellol (CIt), 1,8-cineole (CIn), limonene (LI), and eugenol (EU) computed from the chemical structure and the canonical smiles, obtained from the PubChem database [27] and calculated with the web tools SwissADME [28] and pkCSM-pharmacokinetics [29].

Computed property	CIt	CIn	LM	EU
Molecular Weight (MW, g/mol)[a]	156.26	154.25	136.23	164.20
XLogP3-AA - Lipophilicity[a]	3.2	2.5	3.4	2
Hydrogen Bond Donor Count (HBDC)[a]	1	0	0	1
Hydrogen Bond Acceptor Count (HBAC)[a]	1	1	0	2
Rotatable Bond Count (RBC)[a]	5	0	1	3
Topological Polar Surface Area (TPSA, Å^2)[a]	20.2	9.2	0	29.5
Complexity[a]	112	164	163	145
Vapor pressure (mmHg at 25 °C)[a]	0.04	1.9	1.55	0.01
Consensus Log $P_{o/w}$ - Lipophilicity[b]	2.92	2.67	3.37	2.25
Log S (ESOL) - Water Solubility[b]	-2.94	-2.52	-3.50	-2.46
Log S (Ali) - Water Solubility[b]	-4.03	-2.59	-4.29	-2.53
Log S (SILICOS-IT) - Water Solubility[b]	-2.21	-2.45	-2.26	-2.79
[2]Blood brain barrier (BBB) permeant[b]	Yes	Yes	Yes	Yes
P-gp substrate[b]	No	No	No	No
Log Kp - Skin permeation (cm/s)[b]	-4.48	-5.30	-3.89	-5.69
Human Intestinal absorption (HIA, %)[c]	92.83	96.505	95.898	92.041
BBB permeability – Distribution (log BB)[c]	0.627	0.368	0.732	0.374
CNS permeability – Distribution (log PS)[c]	-2.222	-2.972	-2.37	-2.007

[a]PubChem; [b]SwissADME; [c]pkCSM-pharmacokinetics; [1]BOILED-Egg (white) and [2]BOILED-Egg (yolk) [28].

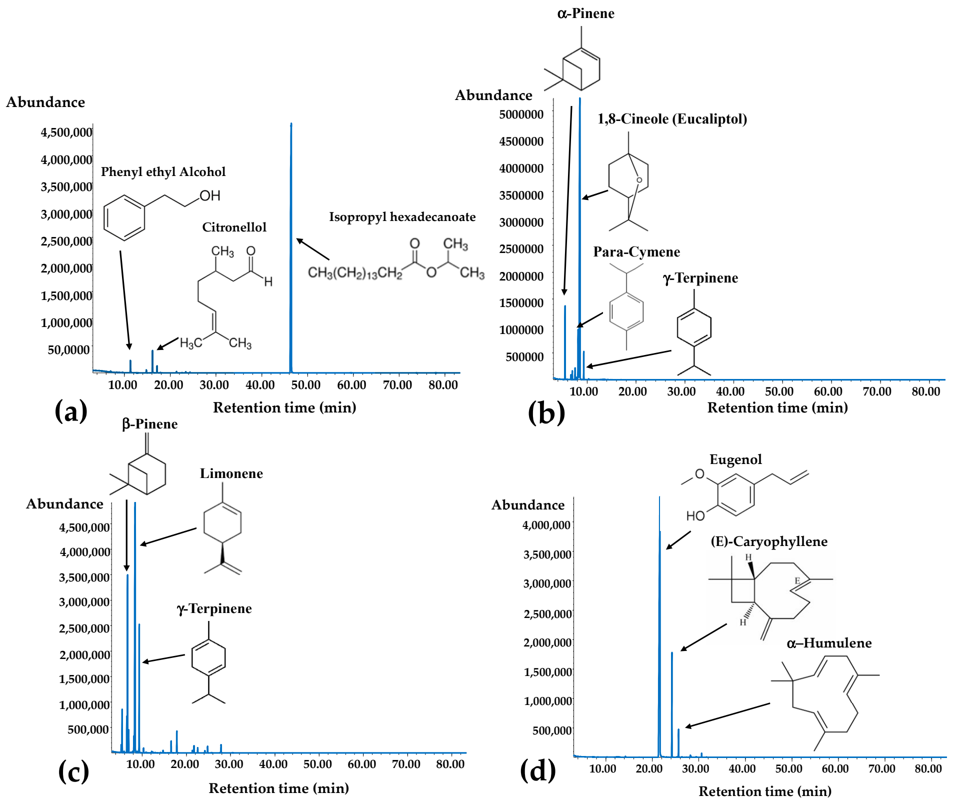

Figure S1. Full chromatograms, obtained by GC/MS technique on an HP-5MS capillary column, of EO1 (a), EO2 (b), EO3 (c), and EO4 (d) with the indication of the main volatile components.

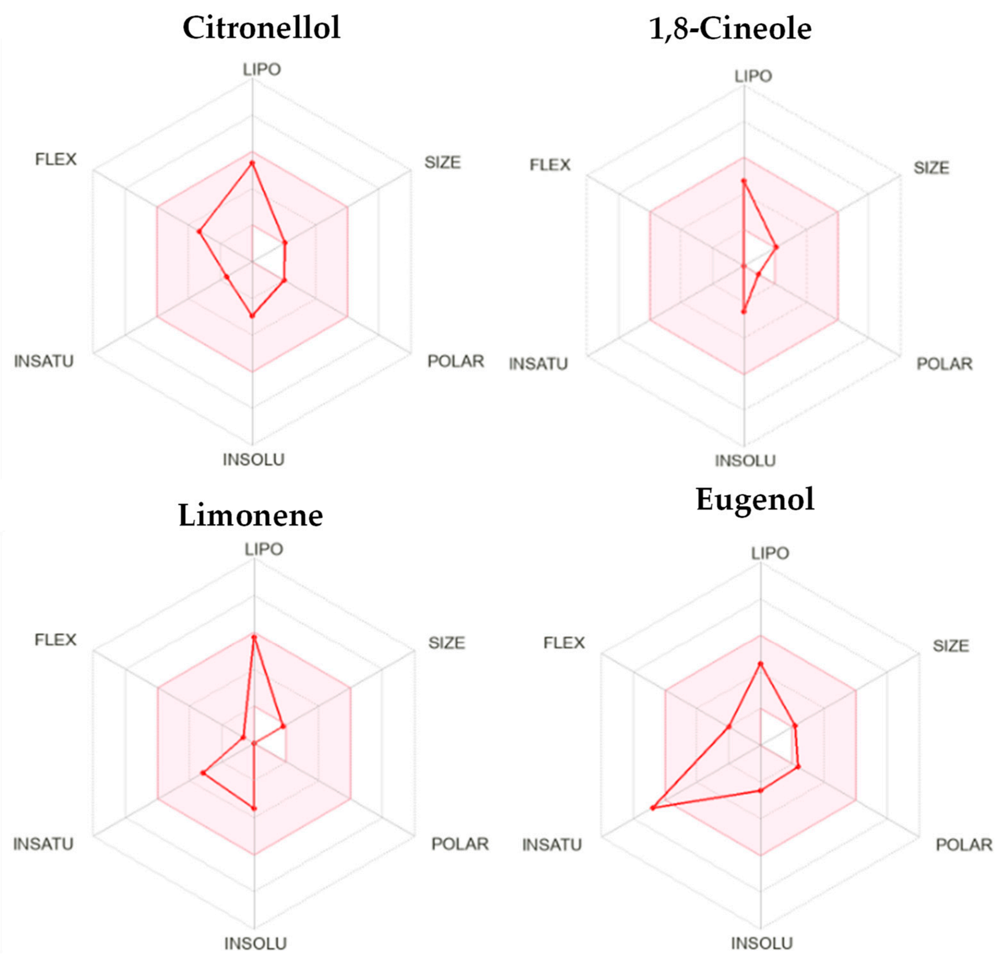

Figure S2. Bioavailability radars of EOs main components citronellol (EO1), 1,8-cineole (EO2), limonene (EO3), and eugenol (EO4) computed from the canonical smiles by the web tools SwissADME [28].

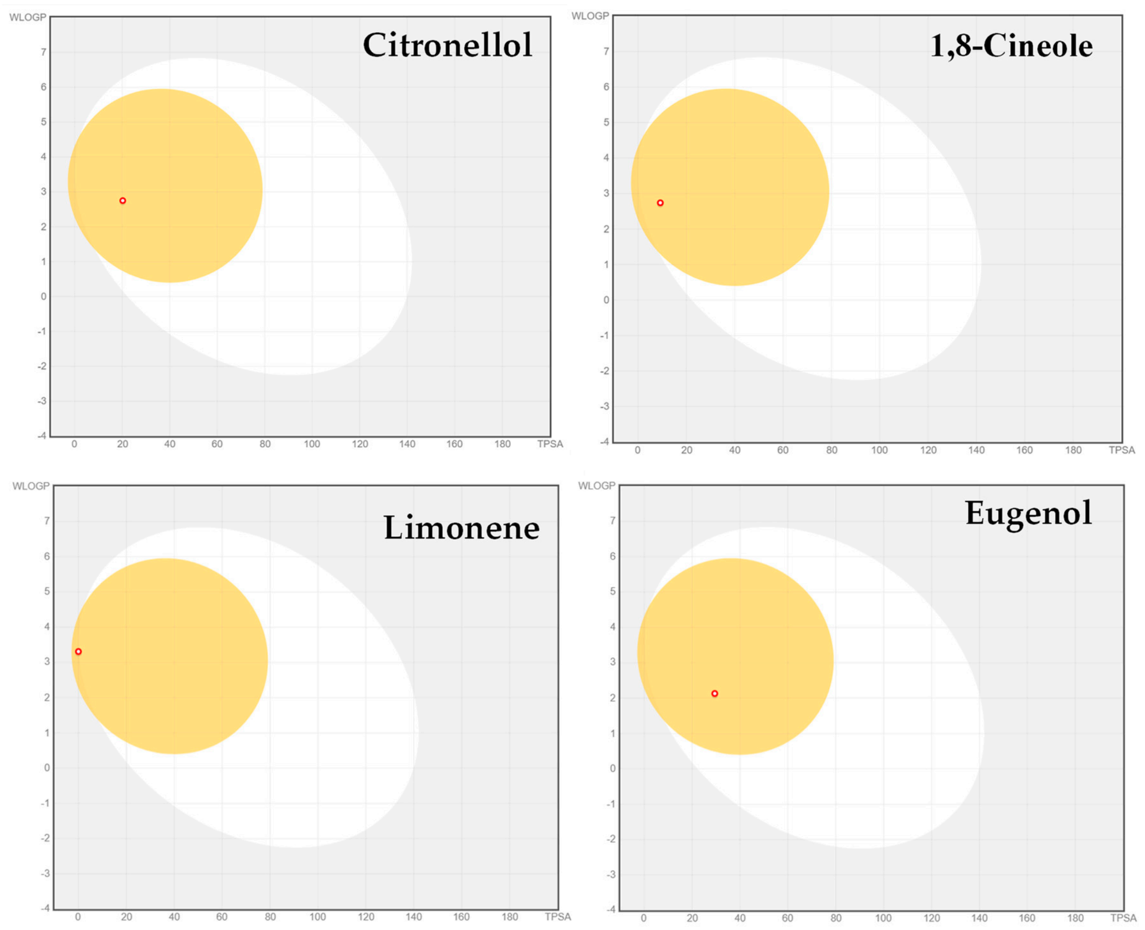

Figure S3. "BOILED-Egg" graphs of main components of EO1 (citronellol), EO2 (1,8-cineole), EO3 (limonene), and EO4 (eugenol) computed from the canonical smiles by the web tools SwissADME [28].
